# Predictors of Adherence to Multiple Clinical Preventive Recommendations among Adults with Diabetes in Spain

**DOI:** 10.1371/journal.pone.0131844

**Published:** 2015-06-29

**Authors:** Isabel Jimenez-Trujillo, Rodrigo Jiménez-García, Jesus Esteban-Hernández, Valentin Hernández-Barrera, Pilar Carrasco Garrido, Miguel A. Salinero-Fort, Juan Cardenas-Valladolid, Ana López-de-Andrés

**Affiliations:** 1 Preventive Medicine and Public Health Teaching and Research Unit, Health Sciences Faculty, Rey Juan Carlos University, Alcorcon, Comunidad de Madrid, Spain; 2 Dirección Técnica de Docencia e Investigación, Gerencia Atención Primaria, Madrid, Comunidad de Madrid, Spain; University of Louisville School of Medicine, UNITED STATES

## Abstract

**Objective:**

This study aims to describe adherence to seven clinical preventive services among Spanish adults with diabetes, to compare adherence with people without diabetes and to identify predictor of adherence to multiple practices among adults with diabetes.

**Design:**

Cross-sectional study based on data obtained from the European Health Survey for Spain 2009 and the Spanish National Health Survey 2011. We analyzed those aged 40-69 years (n= 20,948). Diabetes status was self-reported. The study variables included adherence to blood pressure (BP) checkup, cholesterol measurement, influenza vaccination, dental examination, fecal occult blood test (FOBT), mammography and cytology. Independent variables included socio-demographic characteristics, variables related to health status and lifestyle factors.

**Results:**

The study sample included 1,647 subjects with diabetes and 19,301 without. Over 90% had measured their BP and cholesterol in the last year, 44.4% received influenza immunization, 36.4% had a dental checkup within the year and only 8.1% underwent a FOBT. Among diabetic women 75.4% had received a mammography and 52.4% a cytology in the recommended periods. The adherence to BP and cholesterol measurements and influenza vaccination was significantly higher among those suffering diabetes and cytology and dental checkup were lower. Only 63.4% of people with diabetes had fulfilled half or more of the recommended practices. Female sex, higher educational level, being married or cohabiting, higher number of chronic conditions and number of physician visits increased the adherence to multiple preventive practices. For each unhealthy lifestyle reported the probability of having a higher adherence level decreased.

**Conclusions:**

Acceptable adherence is found for BP and cholesterol checkups and mammography. Unacceptably low rates were found for influenza vaccine, dental care, cytology and FOBT. Moreover, preventive services are provided neither equitably nor efficiently so future research needs to identify individual and organizational factors that allow interventions to reach these subjects with diabetes.

## Introduction

Diabetes mellitus has a considerable impact worldwide [1–6]. As well as the suffering of individuals and their families, diabetes poses a huge economic burden to nations' health care systems [4–6].

Diabetes is a chronic disease that requires complex continuing medical care and patient self-management to reduce the risk of long-term complications [7]. Receipt of multiple recommended preventive care services can prevent or delay diabetes-related acute and chronic complications [7].

The recommended clinical preventive services include, among others, periodic blood pressure measurement and blood lipid profiles. According to the American Diabetes Association and the Spanish Primary Care recommendations, blood pressure should be measured at every routine diabetes visit and a lipid profile should be performed at least annually [7, 8]. All diabetes patients should also receive influenza vaccination and a dental examination once a year [7, 8].

Despite the potential value of these recommended clinical preventive services, studies conducted in Spain and other countries have shown their delivery to be variable and frequently below desirable levels [9–15].

As well as the specific complications of diabetes epidemiological evidence suggests that people with diabetes are also at significantly higher risk than the general population of developing many forms of cancer [16–18]. Researchers have noted an increased risk of colorectal cancer (CRC) and its precursors in people with diabetes [16, 19, 20]. Furthermore, diabetes has been associated with increased mortality and poorer clinical outcomes among persons with CRC [16, 19, 20].

Women with diabetes have an increased incidence of breast cancer, and women diagnosed with this cancer who have preexisting diabetes are at increased risk of breast cancer mortality compared with those without diabetes [16–18, 21, 22].

The relationship between diabetes and risk of cervical cancer remains to be evaluated, but weight gain has been shown to increase the risk of female’s reproductive organs neoplasms, namely, cancers of endometrium, breast, and cervix [16, 23]. Furthermore, cervical cancer mortality is higher in obese women, and this condition is strongly associated with diabetes [23].

Patients with diabetes should be encouraged to undergo recommended age and sex appropriate cancer screenings [7].

Spanish screening guidelines recommend mammography for women aged 50–69 years every 2 years and beginning at 40 years if any condition that increases risk exists [24, 25]. For cervical carcinoma, recommendations include screening with Papanicolaou smear (cytology) for 2 years starting 3 years after women become sexually active, and if both yield normal results, repeat every 3 years up until 64 years of age [24, 25].

Several Spanish autonomous regions have implemented population-based screening programs for colorectal cancer. These programs include men and women aged 50–69 years as their target population using fecal occult blood tests (FOBT) every two years [24, 25].

In Spain, screening programs for CRC, breast, and cervical cancer prevention are established by the Public Health System and provided free of charge to target populations [24, 25].

Studies conducted in different countries, including Spain, have found that adherence to cancer screening tests among people with diabetes is lower than for those without diabetes [26–31].

Several authors have found that factors such as; age, sex, socioeconomic status, educational level, utilization of health services, ethnic group and unhealthy behavioral risk factors are associated with adherence to clinical preventive practices and cancer screening tests among adults with diabetes [9–15, 26–32].

This study aims i) To describe adherence to clinical preventive services among Spanish adults with diabetes including; cancer screening tests (mammography, cytology and FOBT), blood pressure and cholesterol measurement, influenza vaccine and dental examination; ii)to compare adherence with people without diabetes iii) to identify predictor of adherence to these clinical practices among adults with diabetes and IV) to identify the prevalence and the socio-demographic characteristics and lifestyle factors associated with the simultaneous presence of adherence to a higher number of preventive practices in adults with diabetes in Spain.

## Material and Methods

This study is cross-sectional and based on data obtained from the European Health Survey for Spain (EHSS 2009) and the Spanish National Health Survey (SNHS 2011) that cover a representative sample of the Spanish population.

The EHSS 2009 and SNHS 2011 were conducted by the National Statistics Institute (Instituto Nacional de Estadística, INE) under the aegis of the Spanish Ministry of Health & Social Affairs using identical methodology. Subjects were selected by means of probabilistic multistage sampling, with the first-stage units being census sections, the second-stage units, main family dwellings and the final units (individuals) selected by means of random routes and sex- and age-based quotas. Information was collected through computer assisted personal interviews. The EHSS 2009 data collection period was from April 2009 to March 2010, and the SNHS 2011 from July 2011 to June 2012. More information on the methodology of the surveys used is available elsewhere [33, 34].

In order to create all of the variables in our study, questions that were the same in both surveys were used.

The total sample size in the two surveys used was 22,188 individuals aged 16 years or over for the EHSS 2009 and 21,007 individuals aged 15 years or over for the SNHS 2011. For the purposes of this study we selected and analyzed only those aged 40 to 69 years in each health survey so our final study populations were 10,780 from the EHSS 2009 and 10,168 from the SNHS 2011.

We defined people with diabetes as respondents who answered yes to the question, “Have you ever been told by a doctor that you have diabetes?”

The dependent variables included adherence to seven preventive clinical practices. The following questions were used to collect information about these practices:

- “Have you ever undergone a mammography?” Those who answered the question affirmatively were asked a second question: “When was the last time that you had this mammography performed?” Possible answers were “In the last 12 months”, “More than one, but not more than two years ago”, “More than two, but not more than three years ago", "More than three years ago". With this question, subjects who had had a mammography screening test performed in the last two years were classified as compliant. The remaining subjects were classified as non-compliant. Equivalent questions were used to determine cytology and FOB uptake. In the case of cytology women who had received the screening test within the last three years were classified as compliant, for FOB the time period was two years. Mammography and cytology were analyzed for women aged 40–69 and 40–64 years respectively. FOB was analyzed in both sexes in the age group 40–69 years. The age groups used for each test correspond to the target age recommended for each test in Spain [24, 25].

Respondents who answered yes to the question “During the past 12 months, have you had a flu shot?” were defined as having had an influenza vaccination. The questions: Has your blood pressure been measured by a health professional at least once in the last year? And, has your blood cholesterol been measured by a health professional at least once in the last year? Were used to assess adherence to recommended BP and cholesterol checkups. Finally, the dental examination uptake in the last year was analyzed.

To assess a possible clustering of non-adherence to preventive clinical practices we estimated the number to practices that should have been fulfilled for each diabetic subject according to his/her sex and age. This number ranged from four for a man under 50 years to seven for a woman aged 50 to 64 years. We created a new variable “Adherence level” by dividing the number of preventive practices completed by the number of how many should have been fulfilled according to his/her sex and age. Those with a figure under 25% were classified as “poor adherence” those with 25% to <50% as “low adherence”, those from 50% to <75% as “medium adherence” and those with 75% or over as “high adherence”.

The following independent variables were considered:

1. Socio-demographic variables, such as sex, age, marital status, level of education and country of birth. The categories for these variables are shown in Table 1.
2. Variables related to health status are: i) Number of self-declared physician diagnosed chronic diseases, including the following: asthma, COPD, heart or coronary disease, stroke, cerebral hemorrhage, cirrhosis, hepatic dysfunction and cancer. This variable was classified as “None”, “One or two” and “Three or more”; ii) Number of visits to general physician (GP) in the last month. This variable was classified as “None”, “One” and “Two or more”;
3. Lifestyle factors include: current smoking, alcohol consumption (having consumed more than two alcoholic beverages in the last week), physical activity and obesity (BMI of 30 or more). We classified as “physically active” a subject who self-reported a vigorous or moderate physical activity during at least 30 minutes at a time and at least once a week. Physical activities included; “those you do at work, as part of your house and yard work, to get from place to place, and in your spare time for recreation, exercise or sport.” Those who did not fulfill this level of activity were classified as having a sedentary lifestyle.

**Table 1:** Distribution of the study population by diabetes status and according to study variables.

Variable	Categories	Non Diabetes patients N (%) [CI95%]	Diabetes patientsN (%) [CI95%]	P-value^a^
Sex	Female	10330(53.5) [52.4–57.2]	744(45.2) [42.7–47.6]	<0.001
Age	Mean (SD)	52.9(8.6) [52.8–53.1]	59.2(7.2)[58.8–59.5]	<0.001
Age groups	40–49 years	7900(40.9)[40.2–41.6]	188(11.4) [9.97–13.04]	<0.001
	50–59 years	6154(31.9) [32.2–32.5]	561(34.1) [31.8–36.4]	
	60–69 years	5247(27.2) [26.6–27.8]	898(54.5) [52.1–56.9]	
Marital status	Married or cohabiting	12964(67.2) [66.6–67.9]	1100(66.8) [64.5–69.0]	0.184
Educational level	Primary	10594(54.9) [54.2–55.6]	1266(76.9) [74.7–78.8]	<0.001
	Secondary	5267(27.3) [26.7–27.9]	267(16.2) [14.5–18.1]	
	University	3428(17.8) [17.2–18.3]	114(6.9) [5.8–8.2]	
Nationality	Immigrant	1433(7.4) [7.0–7.8]	82(5.0) [4.0–6.1]	0.706
Number of chronic conditions	None	16158(83.7) [83.2–84.2]	1086(65.9) [63.6–68.2]	<0.001
	1 or 2	3054(15.8) [15.31–16.3]	508(30.8) [28.7–33.1]	
	3 or more	89(0.5) [0.4–0.6]	53(3.2) [2.5–4.2]	
GP visits in last month	None	13634(70.6) [69.9–71.3]	803(48.8) [46.3–51.2]	<0.001
	Once	4467(23.1) [22.5–23.7]	628(38.1) [35.8–40.5]	
	Two or more	1200(6.2) [5.9–6.6]	216(13.1) [11.6–14.8]	
Current smoking	Yes	5739(30.1) [29.4–30.7]	361(22.2) [20.2–24.3]	0.007
Alcohol consumption	Yes	9615(50.5) [49.7–51.2]	621(38.2) [35.9–40.6]	<0.001
Sedentary lifestyle	Yes	13228(68.7) [68.1–69.4]	1233(75.1) [73.0–77.2]	<0.001
Obesity	Yes	3256(17.8) [17.3–18.4]	627(41.6) [39.1–44.1]	<0.001
Number of Unhealthy lifestyle factors	None	1964(10.2) [9.8–10.6]	132(8.0) [6.8–9.4]	0.017
	One	6665(34.5) [33.9–35.2]	557(33.8) [31.6–36.1]	
	Two	7169(37.1) [36.5–37.8]	632(38.4) [36.0–40.7]	
	Three or more	3503(18.1) [17.6–18.7]	326(19.8) [17.9–21.8]	
Mammography	Yes	7438(72.0) [71.1–72.9]	561(75.4) [72.2–78.4]	0.071
Cytology	Yes	6231(67.4) [66.5–68.4]	294(52.4) [48.3–56.5]	<0.001
FOBT	Yes	646(5.7) [5.3–6.1]	118(8.1) [6.8–9.6]	0.158
Influenza vaccine	Yes	3569(18.5) [17.9–19.1]	732(44.4) [42.0–46.9]	<0.001
Dental checkup	Yes	8745(45.3) [44.6–46.0]	599(36.4) [34.1–38.7]	<0.001
BP measurement	Yes	14064(72.9) [72.2–73.5]	1517(92.1) [90.7–93.3]	<0.001
Cholesterol measurement	Yes	13462(69.7) [69.1–70.4]	1496(90.8) [89.3–92.1]	<0.001

FOBT: Fecal occult blood test. BP: Blood pressure.

^a^ P value for differences between diabetic an non diabetic samples. For lifestyle factors and clinical preventive practices the proportions were compared using logistic regression models adjusted by age and sex when required. The statistical power for all analyzes conducted was 80%.

### Statistical analysis

Firstly, a descriptive analysis of all study variables was performed according to diabetes status. The age and sex adjusted adherence to the preventive practices was compared between those with and without diabetes. Secondly, we estimated the adherence to each of the clinical preventive practices studied according to the independent variables for people with diabetes. Thirdly, in order to estimate the independent effect of the study variables on adherence to each of the preventive practices and to assess the clustering of non-adherence to clinical preventive recommendations we analyzed the association for each category of the variable “adherence level” using multivariate analysis.

The bivariate association between variables was assessed using the chi-square test for proportions and the t student test for means.

For multivariable statistical analyses we constructed non-conditional multiple logistic regression and multinomial logistic regression models.

The variables included in these models were those significantly associated in the bivariate analysis and those considered to be relevant in literature. The results of the logistic regression are shown as adjusted OR with their corresponding confidence intervals at 95%.

For each variable, those with unknown or missing responses were excluded.

The statistical analysis was conducted using the STATA 9.1 program, assuming a significance level of α < 0.05 (two-tailed). The statistical power for all analyzes conducted was 80%. We incorporated the survey sampling design and sampling weights.

### Ethic statements

In accordance with Spanish legislation, there is no need for Ethics Committee approval, since the database was obtained from the Spanish Ministry of Health, Social Services and Equality webpage, where it is publicly available. The data was provided to us and therefore analyzed anonymously.

## Results

### Characteristics of the study sample

The study sample included 1,647 (prevalence 7.9%) subjects aged 40 to 69 years who had doctor-diagnosed diabetes and 19,301 without the disease. The distribution of the study population by diabetes status and according to study variables is shown in Table 1.

The diabetic population was significantly older than the non-diabetic (59.2 years vs. 52.9 years). 54.8% of subjects with diabetes were men, and among those without diabetes 46.5% were men (p<0.001). The largest proportion of the diabetic sample (76.9%) was comprised of subjects with a ‘primary studies’ level of education with only 6.9% reporting “university studies”, this proportion reached 17.8% among non-diabetics. The prevalence of chronic conditions was higher among persons with diabetes with 30.8% reporting one or two and 3.2% three or more. The equivalent figures for those not suffering diabetes were 15.8% and 0.5% respectively (p<0.001). More than half of the diabetic subjects (51.2%) had visited their GP in the last four weeks with only 29.4% of non-diabetics having done so (p<0.001).

The prevalence of current smoking and alcohol consumption in the last week was lower among those suffering diabetes, whereas sedentary lifestyle and obesity were higher, these differences remained statistically significant after adjusting by age and sex (p<0.001). The number of unhealthy lifestyles was slightly but significantly higher among those suffering diabetes.

### Adherence to clinical preventive practices

When analyzed by type of preventive health practice we found that adherence among diabetic subjects varied by specific activity. An overwhelming majority (>90%) had measured their blood pressure and cholesterol in the last year, 44.4% received influenza immunization, 36.4% had a dental checkup within the year and only 8.1% underwent a FOBT. Among diabetic women 75.4% had received a mammography and 52.4% a cytology in the recommended period.

As can be seen in Table 1 after adjusting by age and sex, when required, the adherence to blood and cholesterol measurements and influenza vaccination was significantly higher among those suffering diabetes. On the other hand cytology and dental checkup were lower. No significant differences were detected for mammography or FOBT.

### Factors associated to uptake of clinical preventive practices among diabetic patients

The observed adherence to clinical preventive recommendations among diabetes patients according to study variables is shown in Table 2. Diabetic women showed higher adherence than men for BP measurement with no difference according to sex in any other practice. For BP, cholesterol checkups and influenza vaccine adherence increased with age. In mammography the highest uptake was found in the 50–59 years age group and for cytology and dental check up in the youngest group. Married or cohabiting diabetic patients showed higher uptakes for mammography, FOBT and dental checkup. Educational level was positively and significantly associated with the uptake of cytology and dental care checkup. Immigrants reported lower adherence to mammography, cholesterol checkup and influenza vaccination. Suffering more chronic conditions was associated to higher adherence for all the preventive practices except mammography and cytology. For dental care checkups the relationship was reversed, with lower uptake among those with more conditions. A higher number of GP visits was significantly associated to a greater uptake of FOBT, BP and cholesterol checkups.

**Table 2:** Adherence to clinical preventive recommendations among diabetes patients according to study variables.

Variable	Categories	Mammography % (95%CI)	Cytology % (95%CI)	FOBT % (95%CI)	BP checkup % (95%CI)	Cholesterol checkup % (95%CI)	Influenza vaccine % (95%CI)	Dental care checkup % (95%CI)
Sex ^d^	Male	NA	NA	9.2(7.4–11.4)	90.7(88.6–92.4)	90.4(88.3–92.1)	45.7(42.5–49.0)	34.7(31.6–37.8)
	Female	75.4(72.1–78.4)	52.4(48.2–56.5)	6.8(5.1–8.9)	93.8(91.8–95.3)	91.4(89.2–93.2)	42.9(39.4–46.5)	38.4(35.0–42.0)
Age ^a^ ^,^ ^b^ ^,^ ^d^ ^,^ ^f^ ^,^ ^g^	40–49 years	43.2 (32.9–54.2)	75.3(64.8–83.5)	0.0(0.0–0.0)	88.8(83.5–92.6)	86.7(81.0–90.9)	25.0(19.3–31.7)	50.0(42.9–57.1)
	50–59 years	81.5(75.9–85.9)	55.2(48.7–61.5)	7.5(5.6–10.0)	89.5(86.6–91.8)	90.4(87.6–92.6)	30.8(27.1–34.8)	38.2(34.2–42.2)
	60–69 years	78.2(74.0–81.8)	42.3(36.3–48.6)	8.5(6.8–10.5)	94.4(92.7–95.8)	92.0(90.0–93.6)	57.0(53.7–60.2)	32.4(29.4–35.5)
Marital status ^a^ ^,^ ^c^ ^,^ ^g^	Not married	71.1(65.6–76.0)	50.5(43.6–57.3)	5.9(4.2–8.4)	92.1(89.5–94.1)	90.3(87.5–92.5)	44.1(39.9–48.3)	32.7(28.9–36.8)
	Married or cohabiting	78.2(74.1–81.7)	53.5(48.3–58.6)	9.2(7.5–11.1)	92.1(90.3–93.5)	91.1(89.3–92.6)	44.6(41.7–47.6)	38.2(35.3–41.1)
Educational level ^b^ ^,^ ^g^	Primary	75.6(72.1–78.8)	48.7(44.1–53.3)	7.7(6.3–9.4)	92.7(91.2–94.0)	90.9(89.2–92.4)	45.3(42.6–48.1)	32.2(29.6–34.8)
	Secondary	74.7(64.8–82.6)	67.9(57.0–77.2)	9.5(6.3–14.1)	91.0(86.9–93.9)	90.6(86.5–93.6)	38.6(32.9–44.5)	47.6(41.6–53.6)
	University	73.1(53.2–86.6)	70.8(50.1–85.4)	9.2(4.7–17.3)	87.7(80.3–92.6)	90.4(83.4–94.6)	48.3(39.2–57.4)	57.0(47.8–65.8)
Nationality ^a^ ^,^ ^e^ ^,^ ^f^	Immigrant	61.5(45.6–75.3)	48.6(32.7–64.7)	6.5(2.4–15.9)	90.2(81.7–95.0)	84.2(74.6–90.6)	31.7(22.6–42.5)	35.4(25.8–46.3)
	Spanish born	76.2(72.9–79.2)	52.7(48.4–56.9)	8.2(6.8–9.7)	92.2(90.8–93.4)	91.2(89.7–92.5)	45.1(42.7–47.6)	36.4(34.1–38.8)
Number of chronic conditions ^c^ ^,^ ^d^ ^,^ ^e^ ^,^ ^f^ ^,^ ^g^	None	73.8(69.7–77.4)	51.9(46.9–56.8)	5.1(3.8–6.7)	90.0(88.3–91.6)	89.0(86.9–90.7)	40.5(37.6–43.5)	39.3(36.4–42.3)
	One or two	80.5(74.7–85.2)	55.4(47.5–62.9)	12.5(9.8–15.8)	96.5(94.4–97.8)	94.3(91.9–96.0)	50.6(46.2–54.9)	31.7(27.8–35.9)
	Three or more	61.9(40.2–79.7)	27.3(9.0–58.7)	22.0(12.6–35.5)	94.3(83.8–98.2)	96.2(86.1–99.0)	66.0(52.4–77.4)	20.8(11.9–33.7)
GP visits in last month ^c^ ^,^ ^d^ ^,^ ^e^	None	76.9(72.0–81.1)	51.7(45.7–57.7)	6.4(4.8–8.5)	89.5(87.2–91.5)	87.8(85.3–89.9)	39.0(35.7–42.4)	37.1(33.8–40.5)
	One	74.1(68.7–78.8)	52.6(45.8–59.2)	9.0(6.9–11.7)	93.6(91.4–95.3)	92.2(89.8–94.1)	47.1(43.2–51.0)	35.2(31.5–39.0)
	two or more	74.6(65.9–81.6)	54.2(43.4–64.6)	11.3(7.6–16.5)	97.2(93.9–98.7)	98.2(95.2–99.3)	56.9(50.2–63.4)	37.0(30.8–43.7)
Current smoking ^a^ ^,^ ^f^	No	76.6(73.1–79.7)	51.3(46.7–55.9)	8.1(6.6–9.8)	92.3(90.7–93.7)	91.0(89.3–92.5)	47.0(44.2–49.7)	36.8(34.2–39.5)
	Yes	67.6(58.2–75.7)	58.3(48.5–67.3)	7.6(5.0–11.2)	91.1(87.7–93.7)	90.0(86.5–92.7)	35.5(30.7–40.5)	34.9(30.1–40.0)
Alcohol consumption ^d^	No	73.8(0.1–77.2)	50.5(45.8–55.1)	7.3(5.8–9.2)	94.0(92.4–95.3)	91.0(89.1–92.6)	43.5(40.4–46.5)	43.3(40.4–46.3)
	Yes	81.0(73.6–86.7)	60.2(50.9–68.9)	9.0(6.9–11.7)	88.9(86.2–91.1)	90.3(87.7–92.4)	45.9(42.0–49.8)	41.4(37.6–45.3)
Sedentary lifestyle	No	76.1(69.7–81.5)	57.1(49.4–64.6)	7.5(5.2–10.7)	93.1(90.2–95.2)	92.4(89.4–94.6)	46.1(41.3–50.9)	38.7(34.1–43.5)
	Yes	75.2(71.4–78.7)	50.3(45.3–55.2)	8.3(6.8–10.1)	91.7(90.0–93.1)	90.4(88.6–91.9)	43.9(41.1–46.7)	35.4(32.8–38.2)
Obesity ^g^	No	74.4(69.6–78.6)	56.6(50.8–62.2)	7.8(6.1–9.9)	91.5(89.5–93.2)	90.5(88.3–92.2)	43.7(40.4–46.9)	40.0(36.8–43.3)
	Yes	78.3(73.1–82.7)	50.7(43.9–57.5)	8.9(6.8–11.6)	92.3(90.0–94.2)	91.2(88.7–93.2)	46.7(42.9–50.6)	32.7(29.1–36.5)

NA: Not adequate, FOBT: Fecal occult blood test. BP: Blood pressure.

^a^ Significant association for mammography

^b^ Significant association for cytology_;_

^c^ Significant association for FOBT

^d^ Significant association for BP checkup

^e^ Significant association for cholesterol checkup

^f^ Significant association for influenza vaccine

^g^ Significant association for dental care checkup

The statistical power for all analyzes conducted was 80%.

It was found that female non-smokers reported higher mammography uptake and for both sexes those who smoked had lower adherence to influenza vaccination. Diabetic patients who had consumed alcohol in the last week were less likely to have a BP checkup and obese subjects to have a dental care checkup.

The results of the multivariable analysis to identify independent predictors for each preventive clinical practice among diabetic patients are shown in Table 3. Diabetic women were more likely to undergo a mammography in the last two years if they were older, born in Spain and married or cohabiting. Being Spanish born increased the uptake of mammography by 2.68 (95% CI 1.26–5.73) compared with immigrants. Positive predictors for cytology tests included being younger and having a higher educational level. Diabetic women who had completed university studies were 2.08 (95%CI 1.04–5.17) times more likely to report cytology than women with primary studies. The only factor independently associated to having a FOBT or BP checkup was having more chronic conditions. A higher number of chronic conditions and visits to a GP increased the uptake of cholesterol checks. Influenza vaccination was positively associated to higher age (OR = 3.66 for those aged 60–69 compared to 40–49 years age group), university studies (OR = 1.61), more associated chronic conditions (OR = 1.24 “one or two” and OR = 2.28 for “three or more”) and more GP visits. Predictors of more dental care checkups included female sex (OR 1.44; 95%CI 1.12–1.85), younger age, being married or cohabiting, higher education and suffering no chronic conditions.

**Table 3:** Predictors of adherence to specific clinical preventive recommendations among diabetes patients.

Variable	Categories	Mammography OR (95%CI)	Cytology OR (95%CI)	FOBT OR (95%CI)	BP checkup OR (95%CI)	Cholesterol checkup OR (95%CI)	Influenza vaccine OR (95%CI)	Dental care checkup OR (95%CI)
Sex	Male	NA	NA	1	1	1	1	1
	Female	NA	NA	0.70(0.43–1.14)	1.04(0.66–1.62)	0.94(0.62–1.41)	0.79(0.61–1.01)	1.44(1.12–1.85)
Age	40–49 years	1	1	NA	1	1	1	1
	50–59 years	6.30 (3.32–11.97)	0.36(0.19–0.70)	1	0.86(0.49–1.51)	1.30(0.75–2.22)	1.16(0.78–1.75)	0.63(0.44–0.91)
	60–69 years	6.17 (3.30–11.52)	0.25(0.12–0.47)	1.03(0.67–1.59)	1.58(0.88–2.85)	1.31(0.77–2.23)	3.66(2.47–5.43)	0.53(0.37–0.75)
Marital status	Not married	1	1	1	1	1	1	1
	Married or cohabiting	1.68 (1.13–2.51)	1.24(0.83–1.85)	1.52(0.94–2.44)	1.08(0.72–1.64)	1.08(0.73–1.59)	0.95(0.75–1.21)	1.27(1.01–1.61)
Educational level	Primary	1	1	1	1	1	1	1
	Secondary	1.65(0.88–3.10)	1.81(1.02–3.21)	1.42(0.82–2.45)	1.00(0.61–1.65)	1.10(0.68–1.78)	0.93(0.69–1.26)	1.77(1.32–2.36)
	University	1.72(0.61–4.85)	2.08(1.04–5.17)	1.44(0.63–3.24)	0.83(0.44–1.57)	1.16(0.58–2.32)	1.61(1.04–2.49)	2.66(1.75–4.06)
Nationality	Immigrant	1	1	1	1	1	1	1
	Spanish born	2.68(1.26–5.73)	1.72(0.80–3.72)	1.06(0.36–3.14)	1.24(0.56–2.75)	1.57(0.78–3.16)	1.24(0.73–2.11)	1.30(0.78–2.18)
Number of chronic conditions	None	1	1	1	1	1	1	1
	One or two	1.30(0.80–2.10)	1.39(0.88–2.19)	2.10(1.35–3.28)	2.24(1.29–3.89)	1.62(1.00–2.61)	1.27(1.01–1.66)	0.78(0.61–1.01)
	Three or more	0.55(0.19–1.60)	0.48(0.10–2.14)	3.45(1.53–7.79)	1.91(0.44–8.27)	4.18(0.55–31.39)	2.28(1.16–4.48)	0.44(0.22–0.93)
GP visits in last month	None	1	1	1	1	1	1	1
	One	0.77(0.50–1.20)	1.25(0.82–1.90)	1.21(0.76–1.92)	1.33(0.88–2.03)	1.39(0.94–2.04)	1.26(1.00–1.60)	1.05(0.83–1.34)
	two or more	0.66(0.36–1.21)	1.40(0.77–2.52)	1.60(0.90–2.85)	2.25(0.94–5.37)	6.16(1.90–19.96)	1.73(1.21–2.46)	1.24(0.87–1.77)
Current smoking	No	1	1	1	1	1	1	1
	Yes	0.90(0.52–1.59)	1.00(0.60–1.69)	0.88(0.52–1.49)	1.09(0.69–1.73)	1.05(0.67–1.64)	0.78(0.59–1.02)	0.84(0.64–1.11)
Alcohol consumption	No	1	1	1	1	1	1	1
	Yes	1.24(0.73–2.11)	1.40(0.87–2.27)	1.29(0.82–2.01)	0.57(0.38–0.86)	0.97(0.65–1.44)	1.07(0.84–1.37)	1.26(0.95–1.86)
Sedentary lifestyle	No	1	1	1	1	1	1	1
	Yes	0.81(0.52–1.24)	0.73(0.48–1.10)	1.05(0.64–1.71)	0.73(0.46–1.16)	0.72(0.47–1.12)	0.81(0.63–1.04)	0.92(0.72–1.19)
Obesity	No	1	1	1	1	1	1	1
	Yes	1.40(0.93–2.11)	0.87(0.58–1.29)	1.04(0.68–1.58)	0.99(0.67–1.48)	0.98(0.68–1.42)	1.08(0.87–1.36)	0.80(0.64–1.00)

NA: Not adequate, FOBT: Fecal occult blood test. BP: Blood pressure. OR (95%CI): Odds ratios with 95% confidence intervals estimated using logistic regression models. The statistical power for all analyzes conducted was 80%.

Table 4 shows the adjusted odds ratios obtained by multinomial logistic regression for levels of adherence to preventive clinical practices, as the dependent variable, according to study variables and number of unhealthy lifestyle behaviors. Only 18.5% of people with diabetes had fulfilled 75% or more of the recommended practices according to their age and sex and 44.9% ranged between 50% and <75%.

**Table 4:** Predictor of level of adherence to clinical preventive practice recommendations among diabetes patients.

Variable	Categories	Low adherence	Medium adherence	High adherence
Sex	Male	1	1	1
	Female	3.06(1.71–5.49)	3.38(1.90–6.02)	3.33(1.82–6.13)
Age	40–49	1	1	1
	50–59	1.53(0.79–2.97)	1.95(1.01–3.76)	0.68(0.33–1.39)
	60–69	1.54(0.79–2.99)	2.61(1.35–5.06)	1.49(0.74–2.98)
Marital status	Not married	1	1	1
	Married or cohabiting	1.19(0.73–1.94)	1.28(0.79–2.07)	1.72(1.01–2.90)
Educational level	Primary	1	1	1
	Secondary	0.85(0.46–1.57)	1.04(0.57–1.88)	1.77(1.04–3.34)
	University	1.69(0.56–5.09)	3.04(1.04–8.92)	3.89(1.27–11.93)
Nationality	Immigrant	1	1	1
	Spanish born	1.07(0.42–2.76)	1.65(0.63–4.30)	2.19(0.74–6.45)
Number of chronic conditions	None	1	1	1
	One or two	2.38(1.21–4.69)	3.30(1.69–6.43)	3.25(1.62–6.51)
	Three or more	2.77(0.35–7.97)	4.03(0.52–13.96)	5.54(1.69–15.45)
GP visits in last month	None	1	1	1
	One	1.74(1.02–2.93)	1.96(1.17–3.30)	2.13(1.23–3.72)
	two or more	3.53(1.06–11.78)	3.10(0.93–10.29)	6.28(1.85–16.21)
Number of Unhealthy lifestyle factors	Continuous	0.71(0.53–0.93)	0.77(0.58–0.98)	0.68(0.51–0.92)

Reference category “Poor level of adherence” under 25% of recommended practices fulfilled. The statistical power for all analyzes conducted was 80%.

Within each level of adherence to preventive measures, we observed an adjusted OR for female sex of over 3. This means that compared with diabetic patients that had a “Poor adherence level” (reference category) the probability of being in any of the three higher levels analyzed was over three times higher if the patient was a woman. Educational level also showed a significant association with increasingly higher OR as the adherence level rose. This means that higher educational level is strongly associated with a higher adherence to preventive practices. Compared to those in the reference category having university studies increased the probability of having “high adherence” 3.89 times (95% CI 1.27–11.93).

A higher number of chronic conditions and number of physician visits increased the probability of having higher adherence in all the levels analyzed. Finally for each unhealthy lifestyle reported the probability of having a higher adherence level decreased.

## Discussion

Our results suggest that the adherence to recommended clinical preventive practices among diabetic adults included in our study is suboptimal. Only the BP and cholesterol checkup values found can be considered acceptable despite the fact they are below the desirable 100% target.

A study conducted in 2006 in Spain showed that almost 16% and 36% of the diabetic subjects interviewed had not undergone BP and blood lipid measurements in the previous 6 and 12 months, respectively [9]. Current results suggest a possible improvement in the application of cardiovascular risk factor control protocols by primary health care professionals as has been described by other Spanish authors [35–37].

Studies conducted in other countries find adherence levels for these two preventive practices that are similar to ours [11,12, 38, 39]. The diabetes component in the 2011 Survey on Living with Chronic Diseases in Canada—showed that among participants aged 20 years and older with self-reported type 2 diabetes blood pressure was “always or often” measured at diabetes related visits in 89% of patients and blood cholesterol tested less than 3 years ago in 94.3% [12].

We agree with Baillot et al who comments that it appears that BP and cholesterol monitoring have been systematically incorporated into routine clinical care [12].

In our study population we found that only 44.4% of people with diabetes reported an influenza vaccination in the previous year. This figure is remarkably lower than the 60.4% adherence found in Spain in 2006 [9]. Other studies conducted in Spain have detected a significant decrease in high risk groups, including diabetes patients, having an influenza vaccination after the 2009 H1N1 pandemic influenza [40–41]. Data from the US shows higher uptake, in the 2007–2010 national surveys 60% of diabetic participants reported receiving annual influenza vaccinations with a 3.2% increase from the 2003–2006 surveys [11, 38]. The bellow 30% coverage for those aged < 60 years observed in this study is especially worrying. Further studies are necessary to precisely identify reasons for non-compliance and barriers to influenza vaccination among people with diabetes. Meanwhile urgent strategies to improve seasonal vaccination uptake must be discussed and implemented [40].

Adults with diabetes experience an increased oral disease burden, but as found in several studies this may not translate into increased dental care service utilization [14, 42,43].

Our study indicated that individuals with diabetes were less likely to visit a dentist in the last year than age-and-sex adjusted individuals without diabetes (36.4% vs. 45.3%). The use of dental care among Spanish people with diabetes is lower than those described in the US (62.5%) or Finland (63%) [11, 44].

Chaudhari et al used data from the Washington Dental Service (WDS) study and found that patients with diabetes are less likely to use dental services than people without this disease. Furthermore, the group with diabetes had an 18% lower utilization level of overall preventive care including both prophylaxes and periodontal maintenance [42].

In the published literature dental care use is often governed by access factors such as income and dental insurance coverage [45]. Thus, lack of financial coverage could explain the poor uptake of this measure among the oldest and the lower educational level groups in our population, since dental exam is the only preventive activity not delivered by the Spanish National Health System. This finding emphasizes the need to increase awareness and support for oral health care among diabetes patients.

The adherence to breast and CRC screening tests in our study did not differ between those with and without diabetes. The mammography uptake was around 80% for women aged 50 to 69 years with diabetes. Population studies in Spain have found similar adherence to breast screening as those conducted in Sweden, France, the United Kingdom, Holland and Italy, with rates ranging from 70 to 90% [46]. Some, but not all, authors have reported that women with diabetes have lower uptake for mammography than women without diabetes [26–30, 47, 48].

Our results show very low adherence (8.1%) to FOBT among diabetic subjects in Spain. The population-based CRC screening programs have recently been implemented in our country and only cover around 40% of the target population so these facts possibly explain the unacceptably low figures [24, 25, 49, 50]. Data from the US show much higher adherence rates ranging from 38–61% [31, 51]. Previous studies show lower uptake of FOBT among people with diabetes than in non-diabetic subjects, a result not found in our sample [26–28].

The prevalence of cytology over the last 3 years in women with diabetes was 15% lower than in those without diabetes (52.4% vs 67.4%, p<0.001)). The lower adherence among diabetic women has been previously described in Spain and other countries [26, 29, 30, 48].

A possible reason for lower uptake of cancer screening among people with diabetes is that diabetes management may compete with preventative healthcare because of their focus on clinical control of their diabetes and the perception that long-term disease prevention is less important (26, 29, 30, 48, 51]. Physicians and patients tend to prioritize demands and to only deal with the most pressing or symptomatic problems, thus leading to clinical inertia [26, 29, 30, 48, 51].

Among adults with diabetes in our study, several factors have been found to be associated with a higher adherence to specific preventive practices and with a simultaneous uptake of a higher number of preventive practices.

Being married or cohabiting increased the adherence to mammography and dental care and was associated with being in a higher adherence group. These findings suggest that social support is important in order to engage in preventive activities. This support could be instrumental in the form of accompanying someone on a health visit or activity or emotional by discussing concerns around a condition and ongoing care. As a consequence, improved health behaviors and a healthier lifestyle can be developed by the couple [52].

Consistent with existing literature, higher education was associated with the uptake of a greater number of preventive care, specifically cytology, influenza vaccination and dental care; this documents disparity in preventive care by educational status [9, 15, 29, 30, 31, 48, 53].

Educational level, imparts health related knowledge capacity, reflects access to resources including preventive health care, and determines health behaviors, which may explain our findings [15, 29, 30]. These results can imply that stronger public health efforts are needed to increase rates of preventive care uptake for those with lower education levels.

Diabetes patients with more comorbid chronic conditions and more frequent users of GP services were more likely to receive clinical preventive services. This is consistent with the findings in the literature that individuals with multiple conditions are more likely to receive necessary care because they are often seen by more than one provider, and that the primary care provider is now encouraged to not just treat a single disease or condition, but rather to treat the multiple comorbid conditions for overall health management. Previous studies conducted in Spain and elsewhere have also found these associations [9, 26, 29,50].

The association between unhealthy lifestyles and the uptake of specific clinical preventive practices found in our sample showed no conclusive results. However, the multinomial models consistently showed that as the number of unhealthy lifestyles increased the probability of belonging to a group with a higher degree of adherence decreased. Clusters of unhealthy behavioral risk factors have been previously linked to lower uptake of clinical preventive services in both the general population and among diabetic patients in Spain [9, 54]. This relationship is relevant since subjects at highest risk (those with the greatest number of unhealthy behaviors) are less likely to receive the recommended preventive care clinical practices even though they are the ones who would obtain the maximum benefit [9, 54].

Improved diabetes management and uptake of preventive practices will result from complex interactions among factors at the patient level (e.g., motivation), the provider level (e.g., counseling), and the system level (e.g., organization of and patient access to care) [11].

Primary care providers play an important role in increasing participation in clinical preventive services, and this needs particular attention as studies conducted in Spain have shown that physicians’ time to provide preventive services is limited [55].

To increase the proportion of all people with diabetes who receive multiple preventive care services, system-level approaches, such as use of electronic medical records and patient registries, are needed to ensure equity of access and delivery of quality health care [10].

Further research is necessary and should include mixed methods—such as continued population-based surveillance of preventive care services and qualitative research methods—to determine the barriers to preventive practices [10, 11].

We believe that our findings can help inform policy makers developing public health strategies around subpopulations at risk.

Our study has several strengths. We used data from two successive, nationally representative surveys conducted in the Spanish population with diabetes, and were able to collect information for many socio-demographic characteristics and lifestyle behaviors which are not available in medical records. We joined the two databases for the following reason i) The two surveys use identical sampling and interviewing methods and the same questions. The institution which carried out both surveys is the National Statistics Institute (INE) that has conducted all the national surveys done in Spain since 2003. This institution guaranties the highest quality controls. (www.ine.es); ii) The two surveys were conducted over a continuous four year period, from April 2009 to March 2010 the EHSS 2009 and from July 2011 to June 2012 the SNHS 2011. So we think that the time effect, if any, would be of small magnitude; iii) When we compared the distribution of the diabetic population between the two surveys no significant differences were found for socio-demographic variables or for the adherence to any of the clinical preventive practices analyzed between the diabetic patients in both surveys and; iv) finally, we needed to join the two databases in order to obtain a large enough sample size to analyze some dependent variables, such as FOBT, or independent variables, such as educational level or nationality.

However there are some limitations to our study. First, since our study was based on a cross-sectional design, there was no information on the temporal relationship and therefore, causal associations were unable to be made. Second, the validity of the questions used to classify subjects as diabetic or nondiabetic should be evaluated. A study conducted in Spain reported sensitivities over 70% and specificities over 95%, respectively, using medical records as gold standard [56]. Third, another possible limitation of health surveys is that the use of unvalidated self-reported data on unhealthy lifestyle behaviors and preventive care recommendation fulfillment could lead to possible biases, due to recall errors or to the tendency of individuals to give socially desirable responses within interviews [57, 58]. Fourth, other relevant variables such as years since diabetes diagnosis, type of diabetes, insulin use or glycemic control are not collected by the surveys used and may act as confounding factors since some of them have been found to be associated with adherence [15, 31, 50]. We were unable to distinguish between diabetes types which may differentially affect behaviors; however, approximately 95% of Spaniards with diabetes have type 2 diabetes [3, 6]. Unfortunately, we don’t have any personal identifiers (name, date of birth medical records number, social security number …) of the subjects included in the surveys so it is not possible to conduct any additional tests or to find their medical records to confirm the diagnosis of diabetes or to collect information on other relevant chronic complications such as diabetic neuropathies. Fifth, in order to be able to sum the number of preventive clinical practices fulfilled and the number of unhealthy lifestyles for each subject these variables had to necessarily be categorized as “yes/no” and this implies losing detailed information. Sixth, we only included subjects aged 40 to 69 years. This is justified because in Spain 40 years is the recommended age at which the blood pressure control and a lipid profile should be performed at least annually for the general population [54]. Furthermore, this is also the age that the recommendation for mammography starts in Spain [24, 25]. The upper age cut point (69 years) was decided because the recommendation for mammography and FOBT end at this age [24, 25].

Lastly, the initial response rates to the EHSS 2009 and SNHS 2011 were 65% and 61% respectively, so a possible nonresponse bias must be considered [33, 34].

## Conclusions

We conclude that adherence to recommended clinical preventive practices is under desirable levels among Spanish diabetes patients with 36% fulfilling under half of the recommended practices according to their age and sex. Acceptable adherence is found for BP and cholesterol checkups and mammography. Unacceptably low rates, even decreasing over time, were found for influenza vaccine, dental care, cytology and FOBT. Moreover, preventive services are provided neither equitably nor efficiently, since subjects with lower educational level, unmarried or non cohabiting individuals with more unhealthy lifestyle behaviors are less likely to receive them. Future research needs to identify individual and organizational factors that allow interventions to reach these high-risk subjects, minimizing the number of missed opportunities for delivery of educational and preventive health care for all subjects with diabetes.
