# Correction: Predictors of Adherence to Multiple Clinical Preventive Recommendations among Adults with Diabetes in Spain

**DOI:** 10.1371/journal.pone.0160477

**Published:** 2016-07-28

**Authors:** Isabel Jimenez-Trujillo, Rodrigo Jiménez-García, Jesus Esteban-Hernández, Valentin Hernández-Barrera, Pilar Carrasco Garrido, Miguel A. Salinero-Fort, Juan Cardenas-Valladolid, Ana López-de-Andrés

The following information is missing from the Funding section: This study was co-financed by the European Union through the Fondo Europeo de Desarrollo Regional (FEDER, “Una manera de hacer Europa”).
